# Advances in Orthotic and Prosthetic Manufacturing: A Technology Review

**DOI:** 10.3390/ma13020295

**Published:** 2020-01-09

**Authors:** Jorge Barrios-Muriel, Francisco Romero-Sánchez, Francisco Javier Alonso-Sánchez, David Rodríguez Salgado

**Affiliations:** Department of Mechanical Engineering, Energy and Materials, University of Extremadura, 06006 Badajoz, Spain; jorgebarrios@unex.es (J.B.-M.); fjas@unex.es (F.J.A.-S.); drs@unex.es (D.R.S.)

**Keywords:** rapid prototyping, additive manufacturing, orthoses, prostheses, fused deposition modeling, laminated object manufacturing, selective laser sintering

## Abstract

In this work, the recent advances for rapid prototyping in the orthoprosthetic industry are presented. Specifically, the manufacturing process of orthoprosthetic aids are analysed, as thier use is widely extended in orthopedic surgery. These devices are devoted to either correct posture or movement (orthosis) or to substitute a body segment (prosthesis) while maintaining functionality. The manufacturing process is traditionally mainly hand-crafted: The subject’s morphology is taken by means of plaster molds, and the manufacture is performed individually, by adjusting the prototype over the subject. This industry has incorporated computer aided design (CAD), computed aided engineering (CAE) and computed aided manufacturing (CAM) tools; however, the true revolution is the result of the application of rapid prototyping technologies (RPT). Techniques such as fused deposition modelling (FDM), selective laser sintering (SLS), laminated object manufacturing (LOM), and 3D printing (3DP) are some examples of the available methodologies in the manufacturing industry that, step by step, are being included in the rehabilitation engineering market—an engineering field with growth and prospects in the coming years. In this work we analyse different methodologies for additive manufacturing along with the principal methods for collecting 3D body shapes and their application in the manufacturing of functional devices for rehabilitation purposes such as splints, ankle-foot orthoses, or arm prostheses.

## 1. Introduction

Assistive technologies, such as orthotic or prosthetic devices, have existed for many centuries. Orthotic devices have been widely used not only to provide immobilization, support, correction, or protection, but also to treat musculoskeletal injuries or dysfunctions [1]. In the 1970s, new techniques like plastic coating were developed, due to the demand of orthotic devices with a more attractive appearance, by applying a tinted rubber-based plastic film [2], allowing the improvement of orthoses appearance and comfort. In the early 1980s the rise of additive manufacturing technologies (AMT), popularly known as 3D printing technologies in a manufacturing environment, with the introduction of the stereolithography technique, based on the cure of photopolymer resin in thin layers with a UV laser allowed construction of 3D models. In the following years, other AMT were introduced, such as: fused deposition modeling (FDM), laminated object manufacturing (LOM), selective laser sintering (SLS), 3D printing, and variable rapid prototyping (Polyjet Technology), among others.

**Figure 1:**
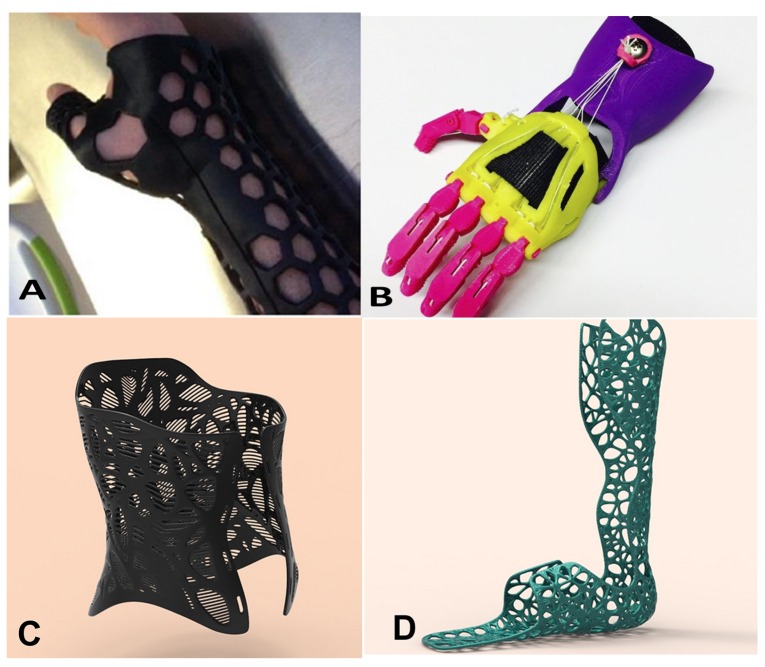
Examples of 3D printed orthotics (**a**) Forearm static fixation (courtesy of Fitzpatrick et al. [21]). (**b**) Cyborg beast hand prosthesis—a low-cost 3D-printed prosthetic hand for children licensed under the CC-BY-NC license (courtesy of Zuniga et al. [19]). (**c**) Spinal brace (courtesy of Andiamo company [22]). (**d**) Ankle-foot orthosis (courtesy of Andiamo company [22]).

The AMTs are included in the field of rapid prototyping techniques (RPT), producing fully functional parts directly from a three-dimensional model without a machining process. A radical change in manufacturing of orthotic devices is already happening due to the exponential growth of RPT over recent decades [3,4]. A quick search in Scopus of 3D printing provides only 122 results before year 2000, 303 results between 2001 and 2005, 756 results from 2006 and 2010, 4521 results for the period 2011–2015, and 22,513 results in the last five years. In the biomedical engineering context, the developments have evolved quickly due to the need for individualized devices able to adapt properly to the patient’s anatomical shapes [5,6,7,8,9]. For this reason, RPT may be helpful in the orthoprosthetic industry, as these devices must adapt perfectly to the body, not only to accomplish their rehabilitative function, but to avoid disuse, as many of these devices produce blistering, ulcers, or discomfort [10,11]. These techniques have been already applied to the manufacturing of spinal braces [11,12], exoskeleton parts [13,14], and passive orthoses [15,16,17], and the application in the medical and dental industry represents one of largest serving industries in the world [18]. Moreover, RPT offer advantages in the design of custom orthotic devices (Figure 1): The orthotic and prosthetic devices are highly customizable, as in Zuniga et al. [19], it is possible to fit the devices to complex geometrical features, with high accuracy, and these devices are manufactured efficiently in terms of cost, lead-time, and product quality [20].

Currently, most rehabilitation devices are designed and hand-crafted by orthopaedists. Therefore, the quality of the product depends on the specialists’ skills and experience [23]. The manufacturing process requires time and depends on the expertise of the specialist to obtain products with functional features that match the unique gait dynamics of each subject. Thus, the need for custom-made products such as orthoses and assistive devices is an explicit need considering the evolution of the technology during the beginning of this century [6,24,25].

**Figure 2:**
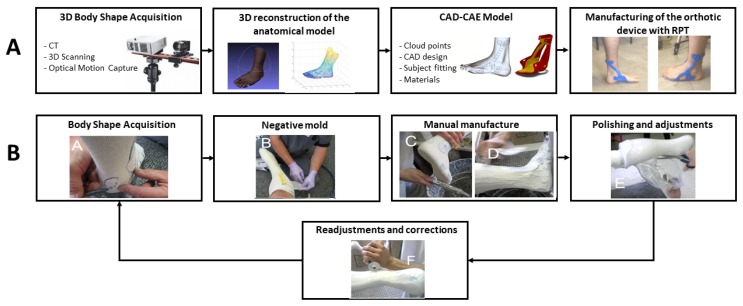
Phases of the manufacturing process of custom-fit orthotic devices. (**A**) Rapid prototyping techniques (RPT) methodology (courtesy of J. Barrios-Muriel). (**B**) Traditional methodology (courtesy of Mavroidis et al. [26] under CC-BY License.) Computer aided design (CAD)-computed aided engineering (CAE), computed tomography (CT).

Regarding orthoprosthetic manufacturing, the first step is to acquire the morphology of the body segment. In the traditional manufacturing process, the subject’s morphology is usually acquired by means of foam or plaster moulds. A prototype is obtained by using a computerized numerical control (CNC) or a milling machine in the thermosetting polyurethane model obtained by the mould. Lastly, the specialist performs several modifications in the device to adjust it to the subject (Figure 2a). However, CNC and milling present some limitations as they are not able to reproduce complex surface designs or to deal with different thickness and materials.

On the contrary, in the new RPT approach, the manufacturing process (Figure 2b) begins with the acquisition of subject’s morphology by means of 3D scanning technologies. Then, computer aided design (CAD)-computed aided engineering (CAE) tools are applied to obtain subject specific designs; whereas, functionality is studied by testing different materials and structures. Lastly, the design is easily exported to an additive manufacturing machine where the prototype is obtained. Manufacturing time may vary between several weeks in the traditional process, to a couple of days in the RPT approach. Therefore, the use of RPT, together with the new 3D acquisition methodologies, represents an alternative in the orthoprosthetic industry.

Many other applications of additive manufacturing (AM) and RPT are in the field of manufacturing of medical instruments [27], drug delivery systems [28], engineered tissues [29,30], scaffolds for bone regeneration [31,32], dental implants [33,34], prosthetic sockets, [35] or surgery [36,37,38]. In this work, we present a review of the developments in the manufacturing of orthotic devices, especially those related to the use of RPT to improve the quality and manufacture times in the rehabilitation field, as in splints, ankle-foot orthoses, or arm prostheses. This review provides comprehensive coverage on the different methodologies ready to be used in the orthotic and prosthetic industry. New 3D data acquisition techniques and the use of different materials are also referred to. This work is intended to be a reference guide on the techniques in this field for practitioners, but also for experienced readers who are interested in pursuing further research.

## 2. 3D Anatomical Data Acquisition Technologies

Applications of RPT combined with different techniques for measuring and modelling the human body are useful to generate new criteria for the orthotic device design. Depending on the data acquisition method used, the data can be expressed as a point cloud, voxels (3D volumetric pixels), or three dimensional coordinates of different anatomical points. Up to date, there is no standardized morphology acquisition procedure, however, there are several acquisition methods to support fabrication using RPT within the field of orthotic devices modelling, including computer tomography, 3D scanning, and different optical motion capture systems.

### 2.1. Computed Tomography

Computed tomography (CT) is a powerful technique to facilitate diagnostics and for surgical planning. Traditionally, the recorded images were in the axial or transverse plane. Currently, modern scanners record images along different planes, enabling volumetric reconstructions for 3D representations. Several studies have applied CT for the manufacture of orthotic devices. For example, Tang et al. [39] recently proposed the use of CT combined with AM techniques to manufacture insoles for diabetes. In their work, they studied pressure and tissue strain along the plantar foot to correlate these variables with the therapeutic effect of footwear and custom-made orthotic inserts, being able to reduce peak plantar pressure by 33.67%. Artioli et al. [40] studied the use of different acquisition techniques to manufacture 3D printed silicone ear prostheses, concluding that the use of CT and AM (using polylactic acid or polylactide, PLA, resolution 100 μm) produce differences of 0.1% between the manufactured prosthesis and the objective model. Liacouras et al. [41,42] used CT to acquire the morphology of the patients stump and to develop the strategies for designing transtibial prosthetic sockets. Moreover, the data of CT allowed a finite element analysis of the prosthetic model socket to calculate the structural stresses and strain at the sockets, as well as the contact pressure at the fibula head. The high image resolution between tissues is one of the greatest advantages of CT, along with the capacity to improve contrast and reduce noise. However, several drawbacks are worthy of mentioning. Radiation is the main concern and the exposition is directly proportional to the duration of the scanning. Other drawbacks are the partial pixel effect, leading to a blurred boundary, as the different densities share common pixels [43].

### 2.2. 3D Scanning

To capture human topography or the external shape, 3D scanning arise as the most practical and comfortable solution. 3D scanning systems use light based techniques to determine the three-dimensional position in space of the different points that integrate the surface of an object. Computer software is then used to reconstruct surfaces from the point cloud and then, the CAD model is obtained.

Currently, 3D scanners for human measurement are available, including the use of single image for reconstruction, structured light technologies, lasers, and different algorithms for stereo reconstruction [44,45,46]. The most common technologies used to reconstruct human body shape are laser and structured light technologies [44]. The laser technique uses a projected laser dot or line from a hand- held device. A sensor measures the distance to the surface, typically a charge-coupled device or a position sensitive device. For static objects, data is collected in relation to an internal coordinate system and, for dynamic conditions, the position of the scanner must be determined to correctly define the point cloud [47]. Structured light methods use a projector-camera system with pre-defined light patterns projected on the moving object. However, a drawback of this technology is the inability to capture certain topography sections of human anatomy with intricate creases and folds, such as between fingers when the hand is in a neutral position, the back of the knee when flexed, or the armpits. The gathered information is more precise, however, and noise is reduced. Recent techniques explore the feasibility of 4D acquisition [48], but to the best knowledge of the authors, there is no report on its use for the design of orthotic and prosthetic devices yet.

Processing time is significantly reduced compared to magnetic resonance imaging (MRI) and CT, as well as the size of the data files [44]. The use of MRI and CT is mostly used to reconstruct of internal organs or tumours with high accuracy for surgical guidance [49]. Recording time and resolution may vary between different 3D scanners, ranging from 3–5 min and a tenth of millimetres for high accuracy systems to a couple of minutes [50] and millimetres for low cost systems [51]. Other advantages of 3D scanning methods are affordable hardware and software, minimal training requirement, availability, accessibility, and efficiency [44].

Several authors suggest the use of reverse engineering software to obtain a refined model by repairing the data. In the pioneering work of Chee Kai et al. [52], a 3D scanning method was selected over the traditional methods for prosthesis modeling, such as plaster-of-Paris impressions, MRI, and CT. Mavroidis et al. [26] used 3D laser scanning to create patient-specific foot orthoses. Surface data of the patient anatomy was manipulated to an optimal form using computer aided design (CAD) software and was fabricated using a rapid prototyping machine. The prototype properly fit the subject’s anatomy compared to a commercial ankle foot orthosis. Paterson [53] investigated the 3D anatomical data acquisition methods to establish a clinically valid, standardized method. He concluded that laser scanning appears to be the most suitable method to reduce the acquisition of ambiguous data and with a high performance in terms of cost, resolution, speed, accuracy, patient safety, cost, and overall efficiency. More recent works, such as those of Mali and Vasistha [54] or Agudelo-Ardila et al. [55], present efficient solutions for the manufacturing of lower and upper limb orthoses, respectively, using reverse engineering.

### 2.3. Optical Motion Capture System

Recently, techniques to measure the topography of the human body in dynamic movements are receiving attention, as the design of orthotic devices should not be designed only for static conditions, as most of them will be used in dynamic conditions to increase rehabilitation. As previously mentioned, there are many commercial 3D systems that are able to measure 3D shapes with high accuracy; however, most of them cannot acquire human motion. An optical motion capture system is a popular technology to capture human movement. These optical systems use several cameras recording in 2D to reconstruct the 3D position of a set of reflective markers placed in anatomical landmarks. These markers should be seen by two or more cameras calibrated to provide overlapped projections. However, the optical motion capture system has a drawback due to the strong limitations related to the number and density of markers [56]. Although only three markers are needed to reconstruct each body segment as a rigid body (e.g., to perform a kinematic analysis), this number is increased if body shape must be also retrieved. The number usually depends on the camera resolution but it does not exceed 60–80 markers per body segment. The main application of the marker-based acquisition technology is in the assessment of the manufactured devices due to the standardized protocols to acquire kinematics [57].

Research has also been focused into structured light using this method [58]. Unkovskiy et al. [59] used a portable structured light scanner to retrieve the topology of the nose cavity and the face to design and manufacture a prosthetic nose. A portable projector was used to project in the region of interest an arbitrary light pattern with a colour code. The system performs the triangulation between the projection pattern and the camera image and to retrieve the correspondence between images. The advantage of this system is the noise reduction compared to the video capture image. The matching of the stereo projection pattern and the camera recorded image is less affected by noise than multiple stereo matching of camera images. Regretfully, synchronous measurement of the entire body shape using multiple projector-camera systems has not been reported yet using this technology. This is essential for capturing the 3D shape motion and to analyse, in the case of gait for example, foot width, length, circumferences, and arch changes during movement [60]. However, there is no commercial system including this technology and thus, more studies should be performed with different methods to compare accuracy and precision between these technologies. The cutting edge technologies in this field are the markerless systems. Chatzitofis et al. [61] proposed recently a low-cost robust and fast system to acquire body kinetics. Although this work still uses reflective straps it could be combined with 4D scanning systems, such as those proposed by Joo et al. [62], to obtain accurate topology and motion of the body segment to rehabilitate.

## 3. Rapid Prototyping Technologies for Orthotic Devices

The combination of CAD and computed aided manufacturing (CAM) is a well-known approach that is receiving increasing attention in the field of orthotic devices to replace the traditional craft practices. As stated by Ciobanu et al. [63], customized manufacturing through RPT requires: 3D scanning of the anatomic surface, 3D surface reconstruction, CAD modeling, conversion to stereolithography format (STL) and, lastly, machining using a special rapid prototyping machine (i.e., a 3D printer) controlled through computer. RPT offer advantages in manufacturing processes of custom-fit orthotic devices, in terms of greater design freedom, ability to create functional elements, superior accuracy and cost efficiency, shorter delivery time, and better user experience of the final product.

In an RPT manufacturing process, a representative virtual 3D CAD model is formed layer upon layer to form a physical object [9]. In RPT, a virtual model of the part is designed through CAD and is converted to a STL file format, which is the default standard file format for RP systems [64,65]. AMT can be categorized in different ways depending on the nature of the fabrication process, such as laser, printer technology, and extrusion technology [7]. There are many different AM processes. Kruth [66] proposed in the early 90’s the use of different methodologies for additive manufacturing, classified according to the material used for the prototype (see Table 1). Nevertheless, Paterson et al. [67] demonstrated that only a few of them could be used to manufacture orthotic and prosthetic devices.

Liquid based process, such as stereolitography (SLA), solid ground curing (SGC), UV light curing (ULC), and ballistic particle manufacturing (BPM); or solid based process, such as laminated object manufacturing (LOM) are methodologies used in the manufacturing of orthoses and prostheses. Nevertheless, the most used methodologies to manufacture orthotic and prosthetic devices are fused deposition modelling (FDM), selective laser sintering (SLS), and powder bed and inkjet head 3D printing (3DP). These methodologies represent an optimized trade-off between cost, delivery time, accuracy, and comfort.

### 3.1. Fused Deposition Modeling (FDM)

**Figure 3:**
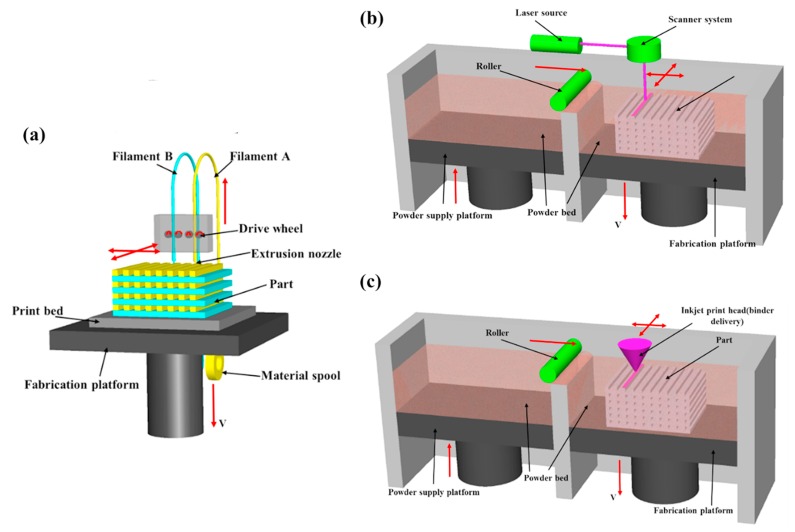
Comparison of the proposed schemes dor rapid prototyping. (**a**) Fused deposition modeling (FDM). (**b**) Selective laser sintering (SLS). (**c**) 3DP. Image adapted from Wang et al. [74] with permission of Elsevier Ltd.

In the FDM process (see Figure 3a), a semi-molten material is extruded through an extrusion head that traverses in the *X* and *Y* axes to create each two dimensional layer of the piece to be manufactured. Two extrusion nozzles compose the movable extrusion head: one to deposit the build material and the other one that contains the support material [68].

In general, the perimeter of each layer is extruded first and then the delimited zone by the previous extrusion is filled by the extruder head by following a pre-defined pattern [68]. Once the layer is completed, the support platform lowers and another layer is extruded. The process continues layer by layer until the piece is complete.

The most common materials for FDM are polycarbonate (PC) and Acrylonitrile butadiene styrene (ABS) or a mixture of them. These materials have similar properties to thermoplastic material for injection moulding [69]. Other materials such as polymers or nylon-based materials may be used. The main advantage of FDM technology is in the use of low-cost materials. Tan et al. [70] were pioneers in the use of used FDM for tibial prosthesis manufacturing and concluded that the functional characteristics of prosthesis were valid for clinical purposes. On the contrary, manufacturing times are high. Since then, an increasing number of applications have arisen for FDM in the biomedical field for upper [17] and lower limb orthoses [50,71], hand prostheses [19], facial prosthesis [40,72], and drug delivery systems [73].

### 3.2. Selective Laser Sintering (SLS)

The DTM Corporation (now a part of 3D Systems) introduced the first SLS system in the 90s [75]. The SLS technique creates three-dimensional solid objects or parts by selectively fusing powdered polymer-based materials such as nylon/polyamide with a CO${}_{2}$ laser, turning powder material into solid objects (Figure 3b). A CO${}_{2}$ laser selectively sinters defined regions by traversing across the powder bed in the *X* and *Y*-axes to form a 2D profile [76]. Once the 2D profile has been completed, the platform lowers, a new layer of powder is distributed and the sintering process is repeated. Subsequently, the process is termed a powder-based fusion process. In general, all materials used are thermoplastics, the most common being polyamide 12 (PA), acrylonitrile butadiene styrene (ABS), and polycarbonate (PC) [77]. These materials lead to a considerable weight reduction improving the usability of the rehabilitation devices.

As an example of the use of this technology in the manufacturing of custom orthoses, Schrank and Stanhope [10] evaluated the accuracy of SLS manufacturing process of ankle foot orthoses (AFO). In this work the discrepancy between the CAD model and final product manufactured with SLS was measured through the Faroarm 3D scanner (accuracy ±25 μm). The results showed values below 1.5 mm (SD = 0.39 mm). Deckers et al. [78] developed and tested an SLS-based AFO, highlighting the need to properly characterize the mechanical characteristics of the AFO such as strength, fatigue, and resistance to impacts. Vasiliauskaite et al. [79] tested a polyamide-based orthoses manufactured with SLS and concluded that the features were similar to a thermoformed polypropylene orthosis, the first one being stiffer than the second but enough for the purpose of rehabilitation.

On the other hand, [80] manufactured a splint for upper extremities using the SLS method. They also concluded that the results of this manufacturing technique were good but did not make any clinical validation of the device. Another similar application of splints using SLS is the design proposed by Evill [81]. In this work, several aspects such as ventilation, hygiene, and aesthetics were improved through CAD. Although there are not conclusive results that confirm these purposes, the new design parameters considerably improve comfort.

### 3.3. Powder Bed and Inkjet Head 3D Printing: 3DP

3DP refers to three dimensional printing, which is understood as the process in which the manufactured product is made by means of powder layers stuck with adhesive. In this process, first, a powder layer is spread on the build platform. Second, a liquid binder is deposited selectively through an inkjet printhead by following a patterned layer in the XY plane. Once the 2D pattern is formed, the platform lowers, the next powder layer is spread and so on. This process is sometimes referred as powder bed and inkjet head 3D printing (or 3DP). It should not be confused with the widespread definition of 3D printing, that involves all additive manufacturing process that result in the manufacturing of tree-dimensional objects. The 3DP process is somewhat similar to SLS (see Figure 3b,c): In 3DP, a printing head places liquid adhesive in the material; whereas, in SLS a CO${}_{2}$ laser is used to fuse the layers. The accuracy of this process is lower than in SLS; nonetheless, this method is preferred due to its low cost and quickness. These qualities have lead 3DP to have a predominant role in the prototyping industry.

The employed materials (mainly thermoplastics as ABS) have the required properties to be used in orthotic and prosthetic applications. Herbert et al. [82] investigated whether this technology was suitable to produce functional prosthesis, and they suggested that, although the manufacturing levels were limited, patients felt more comfortable with prostheses made with 3DP machine (Corporation Z402) than the traditional handmade ones. Regretfully, the resistance was not studied in that work and therefore the durability of the product is unknown. Saijo et al. [83] used this technology to develop patient-specific maxilofacial implants reporting a reduction in operation times. As stated by Ventola [84], 3DP is particularly interesting in tissue engineering and regenerative medicine because of its digital precision, control, and versatility.

To summarize, Table 2 shows a comparison of the different RPT by using the commercial models used in the works described in the bibliography.

## 4. Variable Property Rapid Prototyping

Variable rapid prototyping differs from other AMT as it aims at producing objects of varied properties. In this sense, each material used to manufacture the object provides specific values of strength, strain, heat deflection temperature, etc. [85]. Object geometries recently introduced 3D printers that use polyjet Matrix^TM^ technology to allow the generation of composite material prototypes of varying stiffness and dual material prototypes. The Connex500^TM^ 3D printer operates by using inkjet heads with two or more photopolymer materials. The material is extruded in 16 μm thick layers. Each photopolymer layer is cured by UV light immediately after the extrusion [86].

A carpal skin was produced by Oxman [80], exploring the multiple material building capabilities available with this technology. Campbell et al. [87] explored the benefits of multiple materials integrated into a wrist splint compared to traditional custom-fitted wrist splints of qualified and experienced clinicians. The work focused on the attempt to place multiple materials to behave as hinges or cushioned features as opposed to traditional fabrication processes where a similar approach would be very difficult to replicate. A drawback for this technology is that the actual commercial CAD software is not efficient to apply the design potential and few computation tools manage the physical interaction between material properties. Nevertheless, recent advances in the use of additive manufacturing of hybrid composites, as recently presented for dental implants [88], may lead to a substantial revolution in the field of orthotics, were hybrid exoskeletons are currently changing from rigid structures to wearable garments.

## 5. Material Selection for Orthotic Devices

The choice of material when designing an orthotic device is vital to its success. Physical properties of the orthotic materials include their elasticity, hardness, density, response to temperature, durability, flexibility, compressibility, and resilience [89]. It should be mentioned that each physical property cannot be used alone as a single factor for assessing materials for orthotic devices. A hard material as well as an incorrect aspect in the design may result in an uncomfortable device or a biomechanically detrimental orthotic device. Thermoplastics, composites, and foams are the main materials used to manufacture orthotic devices through AMT [89].

The most known materials used in rapid prototyping manufacturing are ABS (Acrylonitrile butadiene styrene) and PLA (polylactic acid) [90]. ABS is a polymer commonly used to produce car bumpers due to its toughness and strength. PLA is a biodegradable thermoplastic that has been derived from renewable resources such as starch prepared from the grains of corn. These materials are used for the majority 3D printing machines. Rigid and semi-rigid structures can be manufactured with these materials. Depending on the designed thickness, these materials may have different properties.

Soft parts and some semi-rigid parts of orthotic devices are made with foamed materials, usually with open or closed cell structure. The first type allows the movement of gas between the cells whereas the second encloses the gas within the cell walls allowing for a water-tight material. This is desirable for orthotic manufacture as sweat will not be able to penetrate into the material to cause premature degradation. Paton et al. [91] investigated the physical properties of soft materials used to fabricate orthoses designed for the prevention of neuropathic diabetic foot ulcers. They concluded that the most clinically desirable dampening materials tested were Poron^®^96 and Poron^®^4000 (thickness of 6 mm) and the material with the best properties for motion control was ethylene vinyl acetate (EVA). With the evolution of AMT, insoles with variable porous structure and adjustable elastic modulus are being manufactured to adapt to the different needs of patients with diabetes [92].

In the SLS manufacture process, polymer powders and ceramics are mixed to form composites. Rilsan^TM^ D80 DuraForm^TM^ PA and DuraForm^TM^ GF are examples of materials used in SLS techniques. Faustini et al. [93] explored the feasibility of using RPT for AFO manufacturing process. The study determined that the optimal SLS material for AFO to store and release elastic energy was Rilsan^TM^ D80, considering minimizing energy dissipation through internal friction is a desired material characteristic. A relatively recent work from Walbran et al. [94] compares yield stress of custom made orthoses made by SLS of nylon with carbon fibres and FDM with PLA. Although PLA was chosen in terms of mechanical properties and cost, they concluded that the AMT have a strong potential not only to obtain consistent and repeatable models of the subjects affected limb independently of the operator’s skill and restraint of the subject, but to further automate the AFO design process.

Materials used in FDM are those with similar properties to thermoplastic materials for injection moulding. In general polycarbonate (PC) and ABS or their combinations, as mentioned above (PC-ABS, PC-ISO or ABSi), nylon-based materials and other polymers can be used. The main advantage of these materials is the low cost. Finally, in the case of variable properties rapid prototyping base materials, digital materials or composite digital materials can be used. The material properties can be modified by combinations and distribution of different types.

## 6. Discussion

The traditional manufacturing processes for orthotic and prosthetic devices is still mostly hand-crafted and requires special abilities from the othopaedist to obtain a quality product. Nevertheless, this manufacturing process, in general, produces discomfort to the patient. The acquisition of the morphology of the subject is not a clean process as the use of plaster is required to obtain the mould. Additionally, the final product may produce blistering on the subject’s skin, as the morphology is acquired in static conditions.

Alternatively, RPT and AMT have a strong potential to change not only the way in which orthotic and prosthetic products are designed, but also the manufacturing process and the specialist profile. The use of RPT in the orthoprosthetic industry may suppose a considerable change in the know-how; however, it also leads to important benefits. The goal is to accelerate the reconstruction process of 3D anatomical models and biomedical objects for the design and manufacturing of medical products and simulate 3D body shape to design the most suitable orthoses for the patient. Thus, the use of CAD and RPT facilitate the design and fabrication of custom-fit orthotic products with a number of advantages over traditional methods: the use of new materials, customized designs, virtual testing, etc.

The application of these technologies may lead to a significant improvement in the orthotic manufacturing process as production times are lower, morphology acquisition is faster and more pleasant for the patient, as plaster moulds are suppressed and manufacturing errors are minimized. Considerable effort has been applied in the application of AMT to the medical industry and specifically in the design and manufacturing of orthoses and prostheses for rehabilitation purposes [89], to mitigate the effects of aging [95], in the design of active wearable exoskeletons [96], and also to bring this technology closer to the general public [19,97].

The inclusion of these manufacturing methods requires a high investment in equipment, materials, and training that may cause hesitation from investors or orthotic and prosthetic technicians. Moreover, healthcare specialists show some reservations to change their own work routines [86]. Nevertheless, the eruption of 3D printers into the market and the continuous improvements made in this field will give an impulse to the implementation of this technology in the orthoprosthetic industry. In addition, the experience shown in other medical fields, such as in dental implant manufacturing will make possible the implementation of this technology to produce orthoses and prosthesis to reduce waiting lists.

The mass production of these aids must include a series of key points leading to high quality patterns in the manufacturing process. These key points are: acquisition of the subject’s anthropometric data, product design, material selection, manufacturing process planning, and product service, among others. The correct application of these steps reduces the total production time and, therefore, the delivery times. Thus, the inclusion of RPT to produce high quality and short delivery time orthotic and prosthetic devices, that also satisfies functionality and patients comfort is now a reality. Finally, the inclusion of manufacturing technology in a traditional environment such as the orthoprosthetic industry will lead to better products to satisfy the specifications required in rehabilitation.

## 7. Conclusions

In this work, a review of the different RPT applied to the orthoprosthetic industry has been presented. Specifically, the manufacturing process to manufacture orthoses and prostheses have been analysed and the main works in this field have also been presented. These techniques have been shown to have an exponential growth in the following years in the biomedical field. The new advances in the subject’s morphology acquisition as well as the use of RPT can improve the accuracy of the final device, leading to a better rehabilitation process. RPT will help us to optimize the manufacturing process and improve both the design and functionality of assistive devices. Thus, RPT combined with CAD-CAM tools provide a major control in the design and manufacture processes. Finally, the future lines of development in this field will be based on the design of new structures and materials to improve comfort, which will grant the success of the new orthoprosthetic aids.

## Figures and Tables

**Table 1:** Rapid prototyping techniques available for orthoprosthetics.

Material	Process
Liquid base	Stereolithography (SLA)
	Solid ground curing (SGC)
	UV light-curing (ULC)
	Ballistic particle manufacturing (BPM)
Solid base	Laminated object manufacturing (LOM)
	Fused deposition modeling (FDM)
Powder base	Selective laser sintering (SLS)
	3D printing (Polymer injection)

**Table 2:** Characteristics of the most used machines for AMT.

	FDM	SLS	3DP
Model	Dimension STT 768	spro 60 SD SLS	uPrint System
Production Time (h)	7	3	7
Active volume (mm)	203 × 203 × 305	381 × 330 × 457	203 × 152 × 152
Material	ABS P400	Duraform PA (Nylon 12)	ABS P430
Material consumption (g)	40	20.15	55
Cost ($/kg)	190	90	30
